# Medication Adherence and Its Association With Quality of Life Among Hypertensive Patients Attending Primary Health Care Centers in Saudi Arabia

**DOI:** 10.7759/cureus.11853

**Published:** 2020-12-02

**Authors:** Yasir S Alsaqabi, Unaib Rabbani

**Affiliations:** 1 Family Medicine Academy, Qassim Health Cluster, Buraidah, SAU

**Keywords:** hypertension, medication adherence, quality of life, saudi arabia

## Abstract

Background

Chronic diseases require long-term medication and adherence to medication is important for the control of disease as well as prevention of complications. Non-compliance may lead to worsening of the disease, which may affect patients' quality of life. This study aimed to assess the level of medication adherence and its association with quality of life (QOL) among hypertensive patients in Buraidah, Saudi Arabia.

Methods

A cross-sectional study was carried out in which 299 hypertensive patients were recruited from the randomly selected primary health care centers. Medication adherence was assessed by Hill-Bone Medication Adherence Scale, and quality of life was assessed by the World Health Organization’s Quality of Life (WHOQOL)-BREF. Multivariate linear regression was used to assess the association of medication adherence with quality of life. Data was analyzed using SPSS version 21.0 (IBM Inc., Armonk, USA).

Results

The prevalence of poor adherence was found to be 38.8%. We did not find a significant association of medication adherence with any of the four (physical, psychological, social relationship, and environmental) domains of WHOQOL-BREF. However, poor medication adherence was associated with poor perceived overall QOL adjusted β=-0.012 (95% confidence interval [CI]: -0.021 to -0.002; p=0.018) and health adjusted β=-0.013 (95% CI: -0.025 to -0.002; p<0.018).

Conclusion

We found a high prevalence of non-adherence among hypertensive patients. This calls for developing interventions to improve compliance with medications to prevent complications of hypertension. Our study could not find a significant association of medication adherence with any of the domains of QOL, while poor adherence was associated with lower overall perceived QOL and health. Nonetheless, worsening of disease due to non-adherence may affect the QOL of patients. We recommend large scale prospective studies to explore the relationship between medication adherence and QOL.

## Introduction

Chronic diseases are an important public health problem and contribute about 71% of mortality worldwide [1]. In the Kingdom of Saudi Arabia, a large proportion of morbidity and mortality is attributed to chronic illnesses or long-term conditions. Hypertension is a global issue because it is one of the main preventable causes of morbidity and mortality. Hypertension is an illness that has a major effect on communities’ health, and it is widely prevalent in the Arab Gulf region, the Middle East, and the world. It is anticipated to affect around 1.56 billion people worldwide in 2025 [2].

People suffering from long-term conditions receive therapy for a protracted period of time. Quality of life has become an essential measure of results to evaluate the efficiency of the management plan of any illness. Drug therapy alongside with lifestyle adjustments remain the effective control of hypertension, so compliance with the drug is the main factor contributing to attaining the desired clinical result. Non-compliance with antihypertensive drugs is the main cause of poor control of high blood pressure [3].

Studies have shown that adherence rates are typically higher among patients with acute illness compared to those with chronic illnesses [4]. Patients with chronic disorders, particularly asymptomatic conditions, such as hypertension and hypercholesterolemia, are more likely to be non-compliant [5]. Factors that contribute significantly to compliance with medication include lack of knowledge about high blood pressure and its therapy, poor awareness about the importance of adherence, and complex medication regimens [3, 6, 7]. The consequences of medication non-adherence may not only be dangerous for patient’s health but also dramatically increase the financial costs of public health services and it associated with an increase in hospital admissions [8].

Many studies conducted at the global level have reported an important correlation between drug compliance and chronic disease [9, 10]. In Saudi Arabia, only two studies have assessed medication adherence among hypertensive patients [11, 12]. Only one study assessed the association between medication adherence and QOL of patients with chronic diseases within primary care settings [13]. 

Chronic diseases are common, and about 50% of primary care appointments with a physician are due to chronic conditions. It is, therefore, necessary to evaluate the level of adherence to the drugs. This can help improve individuals' compliance with their medications and prevent long-term negative outcomes and attain a better quality of life [14]. Thus, we aimed to assess the level of medication adherence and its relation to the quality of life among adult individuals diagnosed with hypertension visiting primary health care centers in Buraidah, Saudi Arabia.

## Materials and methods

Study design and setting

This was a cross-sectional study conducted at primary health care (PHC) centers in Buraidah, the capital of Al-Qassim Region in the north-central region of Saudi Arabia region with an estimated population of about 590,312 people in 2017. According to the Qassim Directorate of Health Affairs, there are 43 functional primary health care centers in Buraidah city. This study was conducted during the period of one year from 1st July 2019 to 30th June 2020.

Study population and sample size

The study population included patients over 18 years of age visiting PHC centers and a confirmed hypertension diagnosis for at least six months. We used a single proportion formula in the WHO sample size calculator to determine the sample size for this study. We used an expected prevalence of non-adherence of 53% from a previous study [13]. At 95% confidence level and 6% bound on error, the required sample size was 266 hypertensive patients. The sample was inflated to 300 to account for the missing, non-response, or incomplete information.

Sampling procedure

In the first stage, we selected 15 PHCs, which constitute approximately 40% of total centers in Buraidah, by simple random technique. In the next stage, a consecutive sampling strategy was used for patient recruitment. All patients attending the clinics during the study period were asked to participate based on the inclusion criteria. About 10 male and 10 female participants were recruited from each of the selected centers.

Inclusion and exclusion criteria

Patients over 18 years of age, both male and female, with a confirmed diagnosis of hypertension for at least six months were included in the study. Also, the inclusion criteria are taking at least one medication to treat hypertension and ability to communicate in Arabic. Patients with mental health issues, cancers, or any other terminal illness and unwilling to participate in the study were excluded from the study.

Data collecting tools and procedure

Data was collected using a structured questionnaire in Arabic. The questionnaire comprises three sections: socio-demographic and health status, medication adherence scale and WHO Quality of Life (QOL) BREF.

Socio-demographic and health status section has 15 items asking about gender, age, weight, high blood pressure, nationality, educational status, employment status, income, and marital status, number of children, smoking status, and duration of illness, number of comorbidities, and number of current medications.

The Hill-Bone compliance to the High Blood Pressure Therapy Scale is one of two Hill-Bone scales. These are validated questionnaires to assess compliance with medication among hypertensive patients. We used the original, 14-item Hill-Bone scale developed to assess patient behaviors for three important behavioral domains of high blood pressure treatment (the three sub-scales of the original scale): appointment keeping (three items), diet (two items), medication adherence (nine items) [15]. The questions in the scale were translated by a bilingual expert and the first author. Arabic version was then back-translated by another independent bilingual expert who was not involved in the research process. We found a high internal consistency of scale in our study. Cronbach’s alpha for all 14-items was 0.90, while for the medication adherence sub-scale (nine items), it was 0.91.

Higher scores on the adherence scale indicate low compliance [15]. We used four items on the medication subscale which are related directly to the medication adherence (‘‘How often do you miss taking your high blood pressure [HBP] pills when you feel better?’’, ‘‘How often do you miss taking your HBP pills when you feel sick?’’, ‘‘How often do you forget to take your HBP medicine?’’ and ‘‘How often do you miss taking your HBP pills when you are careless?’’) to define an individual as non-adherent. A response of two or higher on adherence scale on at least two of the questions in medication adherence sub-scale was considered non-adherent [16].

We used the World Health Organization’s Quality of Life-BREF (WHOQOL-BREF) to assess hypertensive patients' quality of life. WHOQOL BREF is a validated tool across various population groups and ethnicities. We used a validated Arabic translation of WHOQOL-BREF. WHOQOL-BREF is a generic QOL instrument developed by the WHO to measure QOL in patients with different diseases. WHOQOL-BREF consists of 26 items and assesses QOL across four domains; physical health (seven items), psychological health (six items), social relationships (three items), and environment (eight items). Two remaining questions assessed individuals’ overall perception of QOL, and the other assesses the overall perception of health. All items/questions in the instrument except for question numbers 3, 4, and 26 are scaled positively from 1 to 5 (i.e., high score indicates good QOL) [17]. Negatively phrased items (#3 “To what extent do you feel that physical pain prevents you from doing what you need to do?”, #4 “How much do you need any medical treatment to function in your daily life?”, and #26 “How often do you have negative feelings such as blue mood, despair, anxiety depression?”) were reversed coded before further analysis. The total raw score for these four domains was transformed into a 0 to 100 scale according to the standard procedure defined in WHOQOL user manual. A score of each item was multiplied by four to get scores that are directly comparable to the WHOQOL-100.

Before starting the actual study, we did a pilot study on 10 patients in PHCs which are not part of our study to assess the accuracy of the translation, logical flow of questions, and feasibility of interviews in the waiting area. Minor changes in the questionnaire were made mainly related to formatting errors. Data was collected by the principal investigator from the male participants and by female doctor trained to collect data from female participants through face to face interview. Study purpose was explained to the participants and after assessing their eligibility, patients were invited to participate in the study. Interviews were conducted after taking informed consent.

Statistical analysis

All collected data was entered and analyzed by the Statistical Package for Social Sciences (SPSS) version 21 (IBM Inc., Armonk, USA). Mean with standard deviation for the continuous variables and frequencies and proportions for categorical variables were calculated. Independent sample t-test and one way ANOVA were used to compare adherence and QOL scores among different categories. Univariate and multivariate linear regression analysis was carried out to assess the association between medication adherence scores and QOL. All the variables were assessed in univariate analysis, and those with p-value 0.20 or less in univariate or biologically important were carried forward in the multivariate models. Multicollinearity was checked among the variables using Cramer’s V and phi categorical and correlation coefficient for continuous variables. A value of 0.5 or greater for correlation coefficient was considered for excluding variables from the multivariate model. 

Ethical issues

Ethics approval was obtained from the Qassim Regional Bioethics Committee (Ref # 1440- 1459182). All patients completed written consent forms prior to enrolment in the study. Approval was also sought from the administration of selected PHCs. Informed consent was obtained from all the participants.

## Results

A total of 300 patients were invited to participate in the study period, out of which 299 patients completed the questionnaire. More than half (54%) were female. The mean age was 54.5 (SD=12.68). The majority of patients were of Saudi nationality (93.6%). A little more than half were employed (54.2%), and the mean BMI was 28.09 (SD=4.32). Sixty percent of patients had uncontrolled hypertension. The mean number of current medications was 3.64 (SD=1.98). About 43% of patients had complications, while the mean duration of disease was 11.4 (SD=7.27) years (Table 1). The prevalence of low adherence was found to be 38.8% (n=116).

**Table 1:** Socio-demographic characteristics of hypertensive patients attending PHCCs in Buraidah, Saudi Arabia PHCCs - primary health care centers

Variable	Frequency	Percentage %
Age mean (SD)	54.5 (12.68)	
Gender		
Male	136	45.5
Female	163	54.5
Nationality		
Saudi	280	93.6
Non-Saudi	19	6.4
Blood pressure mean (SD)		
Systolic	145.58 (11.41)	
Diastolic	86.96 (5.42 )	
Uncontrolled	115	38.5
Controlled	184	61.5
Employment status		
Employed	162	54.2
Unemployed	76	25.4
Retired	61	20.4
Educational status		
Uneducated	48	16.1
Up to intermediate	64	21.4
Secondary school	98	32.8
Bachelor and higher	89	29.8
Marital status		
Ever married	264	88.3
Never married	35	11.7
Number of children		
Less than 3 children	75	25.1
3 children and more	224	74.9
Monthly income Saudi riyals (SAR)		
Less than 5000	121	40.5
5000 and more	178	59.5
Smoking status		
Smoker	98	32.8
Non-smoker	201	67.2
BMI		
Weight mean (SD)	74.89 (11.77)	
Height mean (SD)	163.34(6.72)	
BMI mean (SD)	28.09(4.32)	
Comorbidity		
Absent	171	57.2
Present	128	42.8
Duration of disease mean (SD)	11.02 (7.66)	
Number of medications mean (SD)	3.65 (1.97)	
Number of disease mean (SD)	0.65 (0.89)	

In the univariate analysis, we found that age, gender, nationality, blood pressure, employment status, marital status, number of children, comorbidity, duration of disease, number of medications, number of diseases were associated with poor compliance. On the other hand, education and income were associated with good compliance. There was no significant association with body mass index. Associations of sub-scales of Hill-Bone Medication Adherence Scale with various socio-demographic and health-related variables are presented in Table 2. 

**Table 2:** Univariate analysis of factors associated with medication adherence among hypertensive patients in Buraidah, Saudi Arabia SAR - Saudi riyal

Characteristics	Total adherence		Medication		Salt reduction		Appointment	
	B (95%CI)	p-value	B (95%CI)	p-value	B (95%CI)	p-value	B (95%CI)	p-value
Age	0.36 ( 0.30 – 0.41)	<0.001	0.05 ( 0.03 – 0.07 )	<0.001	0.06 ( 0.05 – 0.08 )	<0.001	0.48 ( 0.41 - 0.55)	<0.001
Gender (Female)	- 0.99 ( - 2.65 – 0.665 )	0.102	-0.41 (-1.00 – 0.17 )	0.239	-0.52( - 0.96 - - 0.08)	0.166	-1.92 ( - 4.23 – 0.38)	0.020
Nationality (Non Saudi)	-4.99 ( -8.34- - 1.64 )	0.007	-0.73 ( -1.92 - 0.42 )	0.004	- 0.67 ( -1.58 – 0.22)	0.231	- 6.39 ( - 11.07 - -1.72)	0.141
Blood pressure (Uncontrolled)	6.40 ( 4.87 – 7.94 )	<0.001	2.08 ( 1.53 – 2.63 )	<0.001	1.28 ( 0.85- 1.71 )	<0.001	9.78 ( 7.69 - 11.87 )	<0.001
Employment status								
Employed	1		1		1		1	
Unemployed	5.24 ( 3.55 – 6.92 )	<0.001	0.77 ( 0.09 _ 1.45)	<0.001	0.81 ( 0.32 – 1.30 )	0.027	6.82 (4.46 – 9.19)	<0.001
Retired	9.70 ( 7.88 – 11.52)	<0.001	1.71 ( 0.98 – 2.45 )	<0.001	1.94 ( 1.41 – 2.47 )	<0.001	13.35 ( 1.80 – 15.91)	<0.001
Educational status								
Uneducated	1		1		1		1	
Up to intermediate	-2.36 ( -4.85 – 0.12)	0.083	-4.53 (-1.40 – 0.50)	0.063	- 0.27 (-0.98 - 0.43)	0.347	-3.09 (-6.59 - -0.40)	0.443
Secondary	-6.39 (-8.69 - - 4.09)	<0.001	-1.00 ( -1.87 - -0.12)	<0.001	-0.92 ( - 1.57 - -0.27)	0.026	- 8.32 ( -11.54 - - 5.10)	0.006
College and higher	-8.25 (-10.59 - - 5.91 )	<0.001	-1.57 (-2.46 - -0.68 )	<0.001	- 1.38 ( -2.04 - -0.71)	<0.001	- 11.20 ( -14.48 – 9.19)	<0.001
Marital status (Unmarried/single)	3.84 ( 1.30 – 6.38 )	0.014	0.29 ( -0.61 – 1.21 )	0.003	0.3 ( - 0.37 – 0.99)	0.518	4.45 ( 0.90 – 8.01)	0.373
Number of children (≥3)	4.07 ( 2.21 – 5.92)	<0.001	0.66 ( -0.00 – 1.34 )	<0.001	0.75 ( 0.25 – 1.26 )	0.051	5.49 ( 2.90 - 8.08 )	0.003
Income status (5000 and more SAR)	- 4.32 ( -5.94 - -2.71 )	<0.001	-0.72 ( -1.31 - -0.13 )	<0.001	- 0.70 ( -1.14 - - 0.25)	0.017	-5.75 ( -8.01 - - 3.49)	0.002
Smoking (No)	- 0.20 ( -1.96 – 1.56)	0.305	- 0.43 ( -1.05 – 0.18)	0.820	- 0.65 ( - 1.11 - - 0.18 )	0.169	- 1.28 ( -3.73 – 1.17)	0.006
Comorbidity (Yes)	10.44 ( 9.26 – 11.61)	> 0.001	1.79 ( 1.23 – 2.34 )	<0.001	1.89 ( 1.50 – 2.28 )	<0.001	14.13 ( 12.44 – 15.81 )	<0.001
BMI	- 0.21 ( -0.41 – - 0.23)	0. 080	0.35 ( - 0.32 - 0.10 )	0.28	-0.21 ( - 0.10 - - 0.008 )	0.304	- 0.23( - 0.50 – 0.29 )	0.024
Duration of disease	0.62 ( 0.54 -0.70 )	<0.001	0.09 ( 0.05-0.12)	<0.001	0.12 (0.094-0.14)	<0.001	0.83(0.71-0.95 )	<0.001
Number of medication	2.94 ( 2.69 -3.19 )	<0.001	0.49 ( 0.35 – 0.63)	<0.001	0.54 ( 0.44 – 0.63 )	<0.001	3.97 ( 3.61 – 4.34 )	<0.001
Number of diseases	6.08 ( 5.46- 6.70 )	<0.001	1.13 (0.88-1.44 )	<0.001	1.12 ( 0.91 – 1.34 )	<0.001	8.35 (7.48 – 9.23 )	<0.001

The unadjusted analysis showed that uncontrolled blood pressure, being unemployed, higher income, presence of comorbidity, and duration of disease were associated with higher scores of the physical domain of QOL. Total adherence score was also found to be positively associated with physical QOL. For psychological QOL, education was positively associated. The presence of comorbidity, the number of medications, and the number of diseases were negatively associated with psychological QOL, while compliance was positively associated. In the social relationship domain, female gender, higher education, and income were positively associated. On the other hand, uncontrolled blood pressure, being unemployed or retired, presence of comorbidity, duration, and number of diseases, number of medication and adherence scores were negatively associated with the social relationship domain of QOL. The environmental domain of QOL was negatively associated with uncontrolled blood pressure, number of medications, number of diseases, and total adherence score (Table 3).

**Table 3:** Univariate analysis of factors associated with the quality of life among hypertensive patients in Buraidah, Saudi Arabia SAR - Saudi riyal; QoL - quality of life

Characteristics	Physical health		Psychological health		Social relationship		Environment		Overall perception of QoL		Overall perception of health	
	B (95%CI)	p-value	B (95%CI)	p-value	B (95%CI)	p-value	B (95%CI)	p-value	B (95%CI)	p-value	B (95%CI)	p-value
Age	0.16 (0.09 - 0.22 )	0.184	- 0.13 (-0.28 - 0.02)	0.354	- 0.79(-0.91- - 0.67)	<0.001	- 0.10 ( -0.26 – 0.05 )	0.826	0.002( - 0.16 – 0.16 )	0.980	0.10 ( - 0.08 – 0.29)	0.261
Gender (Female)	0.76 (-2.51 - 0.98)	0.389	1.80 (-2.10-5.70)	0.365	5.26 (1.45-9.08)	0.007	4.39 ( 0.340 – 8.45)	0.034	- 0.02 ( - 0.03 - - 0.02 )	<0.001	- 0.03 ( - 0.04 - - 0.02)	<0.001
Nationality (Non Saudi)	-2.68(-6.24 - 0.87)	0.139	-2.94 (-10.91 - 5.017)	0.467	7.80 ( -0.036 – 15.64)	0.051	- 4.07 (-12.40 – 4.25)	0.336	0.44 ( 0.10 – 0.77 )	0.010	0.50 ( 0.12 – 0.88 )	0.009
Blood pressure (Uncontrolled)	2.85 (1.09 - 4.61)	0.002	- 4.17 (-8.14- - 0.20)	0.400	-10.07 (-13.85 - - 6.28)	<0.001	- 6.42( -10.54 - - 2.30)	0.002	- 4. 33 ( -0.59 - - 0.26 )	<0.001	-0.57 ( - 0.75 - - 0.39)	<0.001
Employment Status												
Employed	1		1		1		1		1		1	
Unemployed	3.07 (0.83 – 5.31 )	0.007	- 4.74 ( - 9.77 – 0.29)	0.065	- 18.60 ( - 23.08 - -14.13)	<0.001	- 3.32 ( - 8.59 – 1.96)	0.217	- 0.77 ( -0.95 - -0.56)	<0.001	- 0.097 ( -1.18 - -0.75)	<0.001
Retired	1.50 ( - 0.57 – 3.58)	0.155	-1.69 ( - 6.34 – 2.96)	0.457	- 10.43 ( - 14.58 - -6.29)	<0.001	0.03 ( -4.85 – 4.93)	0.988	-0.48 ( -0.66 - -0.30)	<0.001	- 0.48 (-0.68 - - 0.28)	<0.001
Educational status												
Uneducated	1		1		1		1		1		1	
Up to intermediate	1.49 ( - 1.37 – 4.35)	0.307	0.30 ( - 6.04 – 6.64)	0.925	1.82 ( -4.00 – 7.65)	0.538	- 0.47 ( -0.071 – 6.16)	0.889	0.26 ( 0.06 – 0.50)	0.045	0.25 ( -0.04 – 0.54 )	0.091
Secondary	-0.97 ( -3.61 – 1.67)	0.471	6.99 ( 1.14 – 12.84 )	0.019	14.40 ( 9.03 – 19.78)	<0.001	7.36 (1.24 -13.48)	0.019	0.75 (0.52 – 0.98 )	<0.001	0.68 ( 0.41 – 0.95 )	<0.001
College and higher	- 1.12 ( -3.81 – 1.56)	0.409	5.54 ( - 0.41 – 11.49)	0.068	15.84 ( 10.38 – 21.30)	<0.001	4.52 ( -1.70 – 10.74)	0.154	0.76 ( 0.52 – 0.99)	<0.001	0.77 ( 0.50 – 1.05 )	<0.001
Marital status (Unmarried/single)	2.05 (-6.44-4.75)	0.135	0.22 (-5.82 - 6.27)	0.941	-1.94 (-7.92 – 4.03)	0.523	1.96 ( -4.36 – 8.28)	0.542	- 0.16 ( - 0.42 – 0.88)	0.196	- 0.16 ( - 0.45 - 0.12 )	0.263
Number of children (≥3)	1.73 (- 0.26 - 3.73)	0.089	2.82 ( -1.65-7.29)	0.215	- 6.84 ( -11.21 - -2.47)	0.002	6.26 ( 1.62 – 10.90)	0.008	- 0.34 ( - 0.52 - - 0.15 )	<0.001	- 0.33 ( - 0.54 - - 0.11)	0.002
Income status (5000 and more SAR)	-1.86 (-3.62 - - 0.09)	0.039	2.81 (-1.13 - 6.76)	0.162	9.65 (5.89 – 13.42)	<0.001	3.60 ( - 0.51 – 7.73)	0.086	0.46 ( 0.30- 0.62 )	<0.001	0.49 ( 0.31 – 0.67 )	<0.001
Smoking (No)	-0.30 (-2.16-1.55)	0.747	0.74 (-3.39 - 4.89)	0.722	2.17 (-1.91 – 6.26)	0.297	3.74 ( - 0.57 – 8.05)	0.089	- 0.10 ( -0.28 – 0.06)	0.226	0.04 ( -0.15 – 0.24)	0.660
Comorbidity (Yes)	1.96(0.22 - 3.71)	0.027	- 4.02 ( -7.9 - - 0.11)	0.043	-16.38( -19.79 --12.97)	<0.001	-3.37 ( -7.42 – 0.71 )	0.106	- 0.81 ( -.095 - - 0.67 )	<0.001	- 0.91 ( - 1.07 - - 0.75)	<0.001
BMI	- 0.03 ( -0.23 – 0.016)	0.729	- 0.12 ( - 0.57 - 0.32 )	0.588	0.14 ( - 0.030 – 0.59 )	0.524	0.09 ( -0.37 - 0.57 )	0.680	0.01 ( - 0.00 – 0.03 )	0.151	0.02( 0.00 – 0.04)	0.016
Duration of disease	0.26 (0.15 – 0.37 )	<0.001	- 0.18 ( - 0.43 – 0.06 )	0.154	-1.11 ( -1.33 - - 0.90 )	<0.001	- 0.21 ( - 0.48 – 0.49 )	0.109	- 0.48 ( -0.05 - - 0.03)	<0.001	-0.05 ( -0.06 - -0.04 )	<0.001
Number of medication	0.88 ( 0.46 – 1.31 )	<0.001	- 1.52 ( -2.48 – 0.55)	0.002	- 4.30 ( -5.13 - -3.46)	<0.001	- 1.46 ( - 2.48 - - 0.043)	0.005	- 0.21 ( - 0.25 - - 0.18 )	<0.001	- 0.26 ( - 0.29 - - 0.22 )	<0.001
Number of diseases	1.67 ( 0.71 – 2.63 )	<0.001	-2.91 ( - 5.07 - - 0.76)	0.008	-8 .89 ( -10 .79 - -6.99)	<0.001	- 2.77 ( - 5.03 - - 0.51)	0.016	- 0.04 ( - 0.54 - -0.39 )	<0.001	- 0.05 ( - 0.66 - - 0.05)	<0.001
Total adherence	0.15 ( 0.07 – 0.24)	<0.001	- 0.24 ( - 0.43 - -0.05)	<0.001	- 0.68 ( 0.85 - - 0.50)	<0.001	- 0.25 ( - 0.45 - - 0.05)	0.011	- 0.03 (-0.04 - - 0.03)	<0.001	- 0.04 ( -0.05 - - 0.03)	<0.001

Table 4 shows the adjusted association of medication adherence with various socio-demographic and health-related variables. We found that uncontrolled blood pressure was associated with higher total adherence scores adjusted β=5.20 (95% CI: 3.50 - 6.50). The presence of comorbidity and duration of disease were also found to be significantly positively associated with total adherence scores adjusted β=9.50 (95% CI: 7.30 - 11.69) and adjusted β=0.23 (95% CI: 0.08 - 0.38), respectively.

**Table 4:** Factors associated with medication adherence among hypertensive patients in Buraidah, Saudi Arabia -- Not included in multivariate models

Characteristics	Total adherence		Medication		Salt reduction		Appointment	
	B (95%CI)	p-value	B (95%CI)	p-value	B (95%CI)	p-value	B (95%CI)	p-value
Age	--	--	--	--	- 0.016 ( -0.47 -0.01)	0.296	--	--
Blood pressure (Uncontrolled)	5.20 ( 3.50 – 6.90)	> 0.001	1.77 ( 0.70 – 2.85)	<0.001	1.78 ( 1.19 – 2.37)	<0.001	--	--
Employment status								
Employed	1		1		--	--	--	--
Unemployed	-1.85 ( -4.55 – 0.83)	0.176	-0.96 ( -2.31 – 0.39)	0.163	--	--	--	--
Retired	2.28 ( -0.45 – 5.01)	0.102	0.167 ( -1.43 – 1.76)	0.837				
Educational status								
Uneducated	1		--	--	--	--	--	--
Up to intermediate	-2.94 ( -5.83 - -0.05 )	0.046	--	--	- 0.38 ( -1.27 – 0.51 )	0.402	--	--
Secondary	-3.05 ( - 6.23 - 0.12)	0.060	--	--	-0.53 ( -1.48 - 0.41)	0.265	--	--
College and higher	-2.74 ( -6.22 – 0.73)	0.121	--	--	- 0.54 ( -1.58 – 0.50)	0.306	--	--
Number of children (≥3)	- 1.35 ( -3.25 -0.53)	0.160	- 0.60 ( - 1.76 – 0.58 )	0.316	--	--	- 0.19 ( -0.67 – 0.28)	0.426
Smoking (No)	--	--	--	--	--	--	- 0.65 (- 1.05 - - 0.25 )	<0.001
Comorbidity (Yes)	9.50 ( 7.30 – 11.69)	<0.001	7.04 ( 5.58 – 8.50)	> 0.001	1.36 ( 0.65 – 2.08)	<0.001	1.16 ( 0.84 – 1.83 )	<0.001
BMI	-0.114 ( -0.206 -.063)	0.206	--	--	0.04 ( - 0.01 - - 010 )	0.183	--	--
Duration of disease	0.23 ( 0.08 – 0.38 )	0.003	--	--	--	--	--	--
Number of medication	--	--	2.09 ( 1.67 – 2.51)	<0.001	--	--	--	--

In the multivariate model, the presence of comorbidity showed a significant negative association with the physical domain of QOL adjusted β=-3.04 (95% CI: -5.71 - -0.37). We did not find a significant association of physical QOL with medication adherence adjusted β=0.09 (95% CI: -0.027 - 0.21). Higher education and the number of children showed a significant positive association with the psychological domain of QOL. There was no significant association of psychological QOL with adherence adjusted β=-0.13 (95% CI: -0.37 - 0.09). Age and presence of comorbidity were negatively associated with social relationship domain of QOL adjusted β=-0.61 (95% CI:-0.80 - - 0.42) and β=-5.78 (95% CI: -10.57 - -0.99), respectively. The secondary level of education and number of children were positively associated. No significant association was observed between total adherence, and social relationship domain of QOL adjusted β=0.12 (95% CI: -0.10 - 0.35). Similarly, no significant association was found between adherence and environmental domain of QOL adjusted β=-0.08 (95% CI: -0.031 - 0.19). Results are presented in Table 5.

**Table 5:** Multivariate analysis of factors associated with the quality of life among hypertensive patients in Buraidah, Saudi Arabia -- Not included in multivariate models QoL - quality of life

Characteristics	Physical health		Psychological health		Social relationship		Environment		Overall perception of QoL		Overall perception of health	
	B (95%CI)	p-value	B (95%CI)	p-value	B (95%CI)	p-value	B (95%CI)	p-value	B (95%CI)	p-value	B (95%CI)	p-value
Age	0.10(-0.03 -0.23)	0.139	--	--	-0.61(-0.80- -0.42	<0.001	--	--	-0.00(-0.01-0.00)	0.136	-0.01(-0.02- -0.00)	0.016
Gender (Female)	--	--	--	--	4.92(1.52-8.31)	0.005	4.95(0.28-9.63)	0.038	--	--	--	--
Nationality (Non Saudi)	-2.00( -5.51- 1.50)	0.262	--	--	3.46(-3.09-10.03)	0.299			0.22(-0.06-0.50)	0.123	0.28(-0.03-0.60)	0.080
Blood pressure (Uncontrolled)	--	--	--	--	-3.22(-6.85-0.41)	0.082	-6.03(-10.66—1.40)	0.011			-0.15(-0.33-0.01)	0.075
Employment Status												
Employed	--	--	1		--	--	--	--	--	--	--	--
Unemployed	--	--	4.39(-2.34-11.13)	0.200			4.04(-3.05-11.15)	0.263	-0.29(0.02-0.57)	0.035	0.34(0.03-0.65)	0.031
Retired			-1.9(-8.21-4.39)	0.551	--	--	1.60(-4.99-8.20)	0.632	-0.06(-0.32-0.19)	0.637	-0.10(-0.39 – 0.18)	0.489
Educational status												
Uneducated	--	--	1		1		1		1		1	
Up to intermediate	--	--	3.80(-3.587-11.20)	0.312	1.69(-3.43-6.81)	0.516	3.55(-3.94-11.05)	0.351	0.27(0.01-0.53)	0.038	0.29(0.01-0.58)	0.042
Secondary	--	--	10.80(2.83-18.77)	0.008	7.00(1.53-12.47)	0.012	13.19(5.12-21.27)	0.001	0.44(0.13-0.74)	0.005	0.25(-0.07-0.59)	0.134
College and higher	--	--	9.80(1.16-18.44)	0.026	4.68(-1.30 -10.66)	0.125	10.50(1.67-19.34)	0.020	0.29(-0.03-0.63)	0.08	0.13(-0.24-0.50)	0.490
Number of children (≥3)	--	--	5.25(0.49-10.01)	0.031	4.66(0.53-8.78)	0.027	8.44(3.59-13.29)	>0.001	--	--	0.16(-0.3-0.35)	0.110
Income status (5000 and more SAR)	-0.52(-2.44-1.38)	0.589	--	--	--	--	--	--	0.20(-0.03-0.43)	0.098	0.30(0.04 -0.56)	0.024
Comorbidity (Yes)	-3.04(-5.71- -0.37)	0.026	--	--	-5.78(-10.57- -0.99)	0.018	--	--	-0.38(-0.60- -0.17)	<0.001	- 0.40( -0.63 -0.16)	<0.001
BMI	--	--	--	--	--	--	--	--	--	--	0.01(0.00-0.03)	<0.001
Total adherence	0.094 (-0.027-0.21)	0.128	-0.13(-0.37-0.09)	0.248	0.127(-0.10-0.35)	0.278	-0.08(-0.031-0.19)	0.653	-0.012 (-0.021- -0.002)	0.018	-0.013 (-0.025- -0.002)	0.018

## Discussion

This study is one of its kind assessing the association of medication adherence and quality of life (QOL and its domains) among hypertensive patients residing in Buraidah, Kingdom of Saudi Arabia. The prevalence of low adherence among hypertensive patients was 38.8% in our study. A facility-based cross-sectional survey reported similar findings, where the prevalence of poor compliance was 39.5% [18]. The most significant factors associated with medication adherence in the study determined were uncontrolled blood pressure, education, any comorbidity, and the duration of the disease.

Evidence suggests that uncontrolled hypertension is significantly associated with poor adherence, which includes refusing to initiate treatment, improper dosage or frequency of medication as prescribed, and non-persistence to therapy for the long-term [19]. Our study reported a similar finding that uncontrolled blood pressure was significantly associated with low adherence, which included compliance with medication as well as salt reduction. Other studies have also reported that non-adherence to medication, a modifiable behavior, was linked with a high rate of blood pressure [20, 21]. The non-compliant behavior for drug adherence could be due to poor health literacy since the relationship of poor knowledge with low medication adherence is found in a few studies [9, 11]. Salt reduction in the diet has been associated with lowering of blood pressure among hypertensive patients [21]. The patients' overall non-adherent behavior might be linked with inconsistent medical care and a careless attitude [20, 22].

Duration of the disease is important in the treatment of chronic conditions. Previous studies have reported duration as a potential factor in the non-adherence of medication [22]. Our study found a negative significant association of disease duration with treatment adherence. Whereas, another study reported a contrasting finding that a shorter duration of less than a year was associated with low adherence [23]. This calls for further in-depth exploration of the reasons behind the duration of the disease and treatment compliance.

We found that the presence of comorbidity was associated with poor adherence. The presence of other chronic diseases along with hypertension and, therefore, multidrug prescription might adversely affect adherence to antihypertensive therapy [19]. A study conducted to assess the association of depression with treatment compliance for hypertension found that poor mental health can adversely cause hindrance with the antihypertensive compliance [24].

Education helps enhance health literacy, as evidence suggested that better education helped achieve a higher level of adherence [25]. Our study result also showed that education is positively associated with adherence to the medication.

The association of low treatment adherence with poor quality of life has been studied less often. However, fewer studies have proven the claim for the linkage of low adherence to the treatment with poor quality of life among hypertensive patients [26, 27]. In contrast, our study did not find a significant association of medication adherence with any of the domains of QOL. However, we did find a significant association with overall perceived QOL and health. Since these two outcomes are based on a single item, therefore caution is needed to interpret the association with medication adherence. The relationship between medication adherence and QOL is a complex mechanism that includes the interplay of various psycho-social variables. These include self-care, knowledge, and attitude towards disease and personal beliefs, negatively impacting disease management, prognosis, and QOL [27].

Our study reported that with the increasing age, the quality of life (the domain of social relationship) becomes poor. Additionally, our findings revealed that the presence of any comorbidity negatively affects the overall quality of life. Previous evidence supports our findings and suggests similar results that aging and co-existing diseases significantly decrease life quality [28]. This could be due to difficulty in managing multiple conditions with poly-pharmacy and dose adjustment with the increasing age in terms of compliance, regular check-ups, and managing drug’ side effects [29].

The association of gender with quality of life has been explored among hypertensive patients where males were found to have a better quality of life, and women were found to be susceptible to physical health [30]. This contrasts with our finding where females have a better quality of life in its social relationship and environmental domain. It could be due to the healthy behavior of women in our study. Controlled blood pressure keeps the person well with the daily routine and functioning, leading to a better quality of life [29]. Our study found that uncontrolled blood pressure is a significant predictor of poor quality of life.

Education and employment have a positive role in a better quality of life, which was suggested by the fact that unemployment and poverty are related to the low quality of life, whereas better education enhances the quality of life in a better way [29]. We also found a similar relationship between unemployment and education with QOL. This could be because education increases the probability of employment and enhances awareness towards the disease, therefore increasing the propensity to pay to seek care and a positive attitude towards disease course.

There are a couple of strengths associated with this study: First, we used validated tools to assess adherence and quality of life which secure internal validity. Secondly, robust statistical methods and multivariate analysis were performed to control confounders and draw conclusions. However, a few limitations need to be considered while interpreting the findings. This study was conducted in one city, which may affect generalizability. Few important confounding variables such as physical activity and biochemical markers such as urinary sodium were not included. Further, self-reporting was used to determine medication adherence, which might result in reporting bias. Compliance could have been checked with empty pill packaging. This, however, was not possible because of limited time and resources. Finally, this was a cross-sectional study; therefore, it is difficult to infer a causal association among variables.

## Conclusions

We found that hypertensive patients in our study had low adherence and poor quality of life. Findings suggest that uncontrolled blood pressure, presence of any comorbidity, and the duration of the disease were associated with low adherence while education up to intermediate was associated with better adherence. Domains of quality of life were not associated with medication adherence. However, overall perceived QOL is negatively associated with low medication adherence. Other significant factors associated with lower QOL in one or more domains were increasing age, uncontrolled blood pressure, and unemployment, and the presence of comorbidity. Education was associated with better QOL. 

Health education and counseling related to disease and its complications, medication adherence, and dietary control such as salt reduction should be mandatory components of hypertension care in PHC settings. This will help achieve better compliance with medication and control of blood pressure, and improved QOL. 
